# Postprandial Plasma Glucose Measured from Blood Taken between 4 and 7.9 h Is Positively Associated with Mortality from Hypertension and Cardiovascular Disease

**DOI:** 10.3390/jcdd11020053

**Published:** 2024-02-04

**Authors:** Yutang Wang

**Affiliations:** Discipline of Life Science, Institute of Innovation, Science and Sustainability, Federation University Australia, Ballarat, VIC 3350, Australia; yutang.wang@federation.edu.au

**Keywords:** non-fasting, postprandial, glucose, diabetes, cardiovascular disease, blood pressure

## Abstract

It is unknown whether postprandial plasma glucose measured from blood taken between 4 and 7.9 h (PPG_4–7.9h_) is associated with mortality from hypertension, diabetes, or cardiovascular disease (CVD). This study aimed to investigate these associations in 4896 US adults who attended the third National Health and Nutrition Examination Survey. Cox proportional hazards models were used to estimate hazard ratios (HRs) and 95% confidence intervals (CIs) of PPG_4–7.9h_ for mortality. This cohort was followed up for 106,300 person-years (mean follow-up, 21.7 years). A 1-natural-log-unit increase in PPG_4–7.9h_ was associated with a higher risk of mortality from hypertension (HR, 3.50; 95% CI, 2.34–5.24), diabetes (HR, 11.7; 95% CI, 6.85–20.0), and CVD (HR, 2.76; 95% CI, 2.08–3.68) after adjustment for all the tested confounders except hemoglobin A_1c_ (HbA_1c_). After further adjustment for HbA_1c_, PPG_4–7.9h_ remained positively associated with mortality from both hypertension (HR, 2.15; 95% CI, 1.13–4.08) and CVD (HR, 1.62; 95% CI, 1.05–2.51), but was no longer associated with diabetes mortality. Subgroup analyses showed that similar results were obtained in the sub-cohort of participants without a prior diagnosis of myocardial infarction or stroke. In conclusion, PPG_4–7.9h_ predicts mortality from hypertension and CVD, independent of HbA_1c_.

## 1. Introduction

Cardiovascular disease (CVD) is the leading cause of death globally, responsible for 17.9 million deaths each year [1]. The global expenditure on CVD ranges between 7.6% and 21.0% of national health expenditures [2]. In the US, CVD costs approximately USD 320 billion per year [3]. Therefore, there is an urgent medical need to identify new risk factors and effective prevention strategies for CVD mortality.

Diabetes affects 8.5% of adults according to the World Health Organization [4]. It is well-known that patients with diabetes have an increased risk of CVD mortality [5,6]. However, the underlying mechanism is not well understood. Postprandial plasma glucose (PPG) is believed to play an important role in diabetes-associated complications [7,8,9]. Therefore, it is of value to investigate the association of PPG with CVD mortality.

To the best of my knowledge, only one study has investigated PPG and CVD mortality [10]. That study found that PPG measured from blood taken between 3 and 7.9 h was positively associated with CVD mortality [10]. However, the PPG measured from blood taken between 3 and 3.9 h did not return to the baseline level and it was higher than PPG_4–7.9h_ [10]. A recent study showed that PPG returned to baseline four hours after a meal regardless of meal type (normal or high carbohydrate) and mealtime (breakfast, lunch, and dinner) [11]. Therefore, the use of PPG measured from blood taken between 3 and 7.9 h is inferior to PPG_4–7.9h_ and the association between PPG_4–7.9h_ and CVD mortality needs to be investigated.

In addition, it has been shown that patients with diabetes have an increased risk of hypertension incidence [12]. However, whether PPG_4–7.9h_ is associated with hypertension mortality or diabetes mortality is unknown.

This study aimed to investigate these unaddressed questions, i.e., whether PPG_4–7.9h_ is associated with hypertension mortality, diabetes mortality, and CVD mortality, using a representative cohort of US adults who attended the third National Health and Nutrition Examination Survey (NHANES III) from 1988 to 1994.

## 2. Materials and Methods

### 2.1. Participants

**Figure 1:**
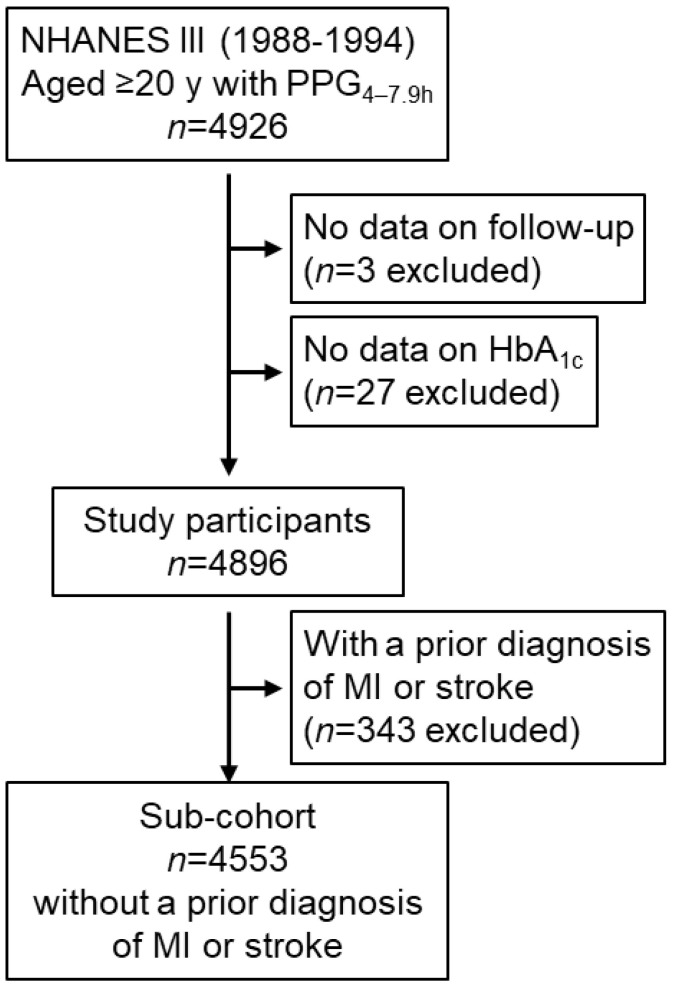
Flow diagram of the study participants. HbA_1c_, hemoglobin A_1c_; MI, myocardial infarction; NHANES III, the third National Health and Nutrition Examination Survey; PPG_4–7.9h_, postprandial plasma glucose measured from blood taken between 4 and 7.9 h.

A total of 4926 adults aged ≥ 20 years who attended the NHANES III recorded postprandial plasma glucose data, measured from blood taken between 4 and 7.9 h. Those who did not have a follow-up time (*n* = 3) or hemoglobin A_1c_ (HbA_1c_, *n* = 27) were excluded. Therefore, the remaining 4896 participants were included in this cohort study, including 343 participants with a prior diagnosis of myocardial infarction or stroke (Figure 1).

### 2.2. Measurement of Plasma Glucose

Plasma glucose was measured using the hexokinase-mediated reaction method, as previously described [13]. In brief, the enzyme hexokinase catalyzed the reaction between glucose and adenosine triphosphate to form adenosine diphosphate and glucose-6-phosphate. In the presence of nicotinamide adenine dinucleotide (NAD), glucose-6-phosphate was oxidized by the enzyme glucose-6-phosphate dehydrogenase to 6-phosphogluconate and reduced nicotinamide adenine dinucleotide (NADH). The increase in NADH concentration was directly proportional to the glucose concentration and was measured spectrophotometrically at 340 nm [14].

### 2.3. Mortality

Data on mortality from CVD (I00–I09, I11, I13, I20–I51, I60–I69), diabetes (E10–E14), and hypertension were directly retrieved from NHANES-linked mortality files [15]. CVD mortality was defined as CVD being listed as the leading cause of death. CVD included ischemic heart disease, heart failure, cardiac arrhythmias, cardiomyopathy, endocarditis, pericarditis, myocarditis, valve disorders, hemorrhage stroke, ischemic stroke, occlusion and stenosis of precerebral or cerebral arteries without resulting in stroke, and other cerebrovascular diseases [10]. Diabetes mortality was defined as diabetes being listed as the leading cause of death. Hypertension mortality was defined as hypertension being listed as an underlying cause of death. The data on hypertension as the leading cause of death were not available.

To evaluate mortality status and the cause of death, the National Center for Health Statistics conducted probabilistic matching [16] to link the NHANES data with death certificate records from the National Death Index (NDI) records, using the following personal identifiers: social security number (nine digits or last four digits), names (first name, middle initial, last name, and father’s surname), date of birth (month of birth, day of birth, and year of birth), state of birth, state of residence, sex, race, and marital status. The NHANES-linked mortality files used the Underlying Cause of Death 113 (UCOD_113) code to recode all deaths according to the International Classification of Diseases, 9th Revision (ICD-9) or the International Classification of Diseases, 10th Revision (ICD-10) for the underlying cause of death [15]. Follow-up time was the duration from the time when the participant was examined at the Mobile Examination Center until death, or until the end of follow-up (31 December 2019), whichever occurred first.

### 2.4. Covariates

Confounding factors included age (continuous), sex (male or female), ethnicity (non-Hispanic white, non-Hispanic black, Mexican-American, or other), body mass index (continuous), education (<high school, high school, >high school, or unknown), poverty income ratio (<130%, 130–349%, ≥350%, or unknown), survey periods (1988–1991 or 1991–1994), physical activity (inactive, insufficiently active, or active), alcohol consumption (never, <1 drink per week, 1–6 drinks per week, ≥7 drinks per week, or unknown), smoking status (past smoker, current smoker, or other), systolic blood pressure (continuous), total cholesterol (continuous), high-density lipoprotein (HDL) cholesterol (continuous), HbA_1c_ (continuous), family history of diabetes (yes, no, or unknown), and fasting time (continuous), as described previously [15,17].

### 2.5. Statistical Analyses

Data were presented as the mean and standard deviation for normally distributed continuous variables, the median and interquartile range for not normally distributed continuous variables, or the number and percentage for categorical variables, to describe the baseline characteristics of the cohort [18]. According to the World Health Organization, 8.5% of adults are affected by diabetes [4]. Therefore, the baseline characteristics of participants were compared between those with PPG_4–7.9h_ in the top decile and those with PPG_4–7.9h_ in the bottom nine deciles. Differences in continuous variables between two groups were analyzed using a Student’s *t*-test (normally distributed), or a Mann–Whitney U test (not normally distributed). Differences among categorical variables were analyzed using Pearson’s chi-square test [19]. The difference in hourly PPG_4–7.9h_ was analyzed using a Kruskal–Wallis one-way ANOVA.

Out of 4896 participants, a total of 115 (2.3%) had missing data, including body mass index (*n* = 14), systolic blood pressure (*n* = 11), total cholesterol (*n* = 53), or HDL cholesterol (*n* = 93). The missing data were imputed via multiple imputation by chained equations, with 20 imputed data sets being created [20]. Little’s test showed that the missing data were not missing completely at random (*p* < 0.001). In all the regression analyses, body mass index, systolic blood pressure, total cholesterol, HDL cholesterol, and HbA_1c_ were natural log-transformed to improve data distribution.

Cox proportional hazards models were used to calculate hazard ratios (HRs) and 95% confidence intervals (CIs) of PPG_4–7.9h_ for mortality from hypertension, diabetes, and CVD [21]. PPG_4–7.9h_ was treated as a continuous variable (natural log-transformed) or a categorical variable. Further analyses were conducted in the sub-cohort of participants without a prior diagnosis of myocardial infarction or stroke.

Sensitivity analyses were conducted when the imputed data were not used, i.e., by excluding those 115 (2.3%) participants with missing data from the analysis, or when those with a follow-up time of <1 year (*n* = 45) or those who were prescribed with insulin or other anti-diabetic medications (*n* = 250) were excluded.

The null hypothesis was rejected for two-sided values of *p* < 0.05. All analyses were performed using SPSS version 27.0 (IBM SPSS Statistics for Windows, IBM Corporation, Armonk, NY, USA) [22].

## 3. Results

### 3.1. General Characteristics

**Figure 2:**
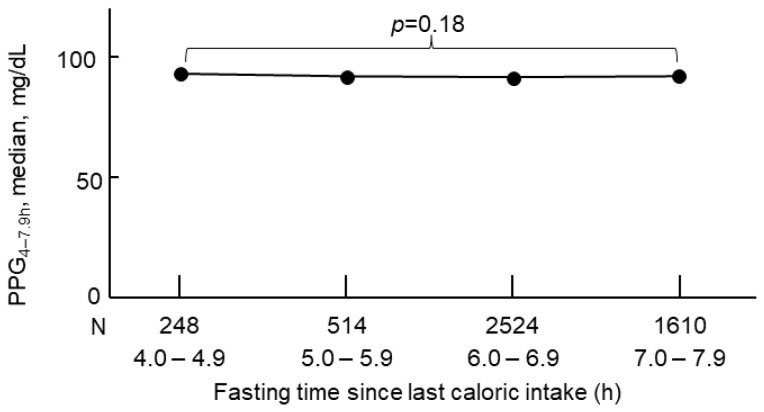
Hourly PPG_4–7.9h_. PPG_4–7.9h_, postprandial plasma glucose measured from blood taken between 4 and 7.9 h.

This cohort included 4896 adult participants with a mean (standard deviation, SD) age of 49 (19) years. Those who had higher PPG_4–7.9h_ were older and had a higher body mass index, systolic blood pressure, and total cholesterol (Table 1). In addition, they were less physically active, had a lower HDL cholesterol, and received less education and income (Table 1). Hourly PPG_4–7.9h_ was similar (Figure 2).

### 3.2. Association of PPG_4–7.9h_ with Mortality

This cohort was followed up for 106,300 person-years, with a mean follow-up of 21.7 years. During the follow-up, 337 hypertension deaths, 70 diabetes deaths, and 835 CVD deaths were recorded.

**Figure 3:**
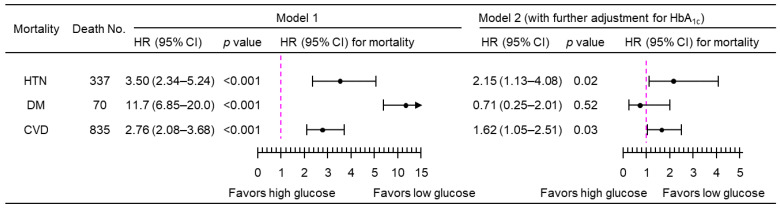
Mortality risk associated with a 1-natural-log-unit increase in PPG_4–7.9h_ in 4896 participants. Model 1: adjusted for age, sex, ethnicity, body mass index, education, poverty income ratio, survey period, physical activity, alcohol consumption, smoking status, systolic blood pressure, total cholesterol, HDL cholesterol, family history of diabetes, and fasting time. Model 2: adjusted for all the factors in Model 1 plus HbA_1c_. CI, confidence interval; CVD, cardiovascular disease; DM, diabetes; HbA_1c_, hemoglobin A_1c_; HDL, high-density lipoprotein; HR, hazard ratio; HTN, hypertension; No., number; PPG_4–7.9h_, postprandial plasma glucose measured from blood taken between 4 and 7.9 h.

**Figure 4:**
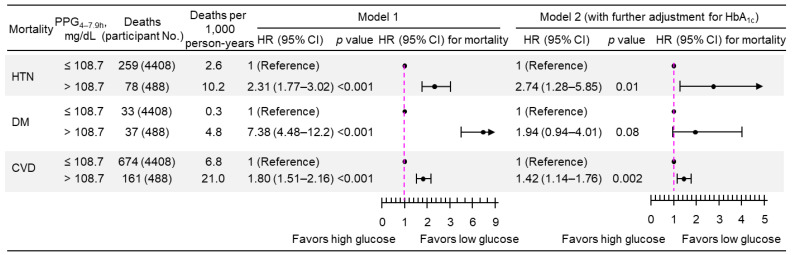
Mortality risk associated with categorical PPG_4–7.9h_ (top decile versus bottom nine deciles) in 4896 participants. Model 1: adjusted for age, sex, ethnicity, body mass index, education, poverty income ratio, survey period, physical activity, alcohol consumption, smoking status, systolic blood pressure, total cholesterol, HDL cholesterol, family history of diabetes, and fasting time. Model 2: adjusted for all the factors in Model 1 plus HbA_1c_. CI, confidence interval; CVD, cardiovascular disease; DM, diabetes; HbA_1c_, hemoglobin A_1c_; HDL, high-density lipoprotein; HR, hazard ratio; HTN, hypertension; No., number; PPG_4–7.9h_, postprandial plasma glucose measured from blood taken between 4 and 7.9 h.

**Figure 5:**
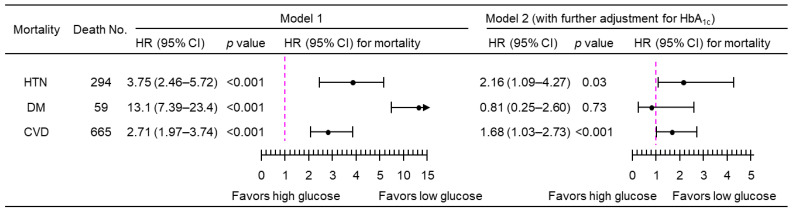
Mortality risk associated with a 1-natural-log-unit increase in PPG_4–7.9h_ in the sub-cohort of 4553 participants without a prior diagnosis of myocardial infarction or stroke. Model 1: adjusted for age, sex, ethnicity, body mass index, education, poverty income ratio, survey period, physical activity, alcohol consumption, smoking status, systolic blood pressure, total cholesterol, HDL cholesterol, family history of diabetes, and fasting time. Model 2: adjusted for all the factors in Model 1 plus HbA_1c_. CI, confidence interval; CVD, cardiovascular disease; DM, diabetes; HbA_1c_, hemoglobin A_1c_; HDL, high-density lipoprotein; HR, hazard ratio; HTN, hypertension; No., number; PPG_4–7.9h_, postprandial plasma glucose measured from blood taken between 4 and 7.9 h.

A 1-natural-log-unit increase in PPG_4–7.9h_ was associated with a higher multivariate-adjusted risk of mortality from hypertension (HR, 3.50; 95% CI, 2.34–5.24), diabetes (HR, 11.7; 95% CI, 6.85–20.0), and CVD (HR, 2.76; 95% CI, 2.08–3.68), after adjustment for all the tested confounders except HbA_1c_ (Model 1; Figure 3). After further adjustment for HbA_1c_ (Model 2, Figure 3), PPG_4–7.9h_ remained positively associated with mortality from both hypertension (HR, 2.15; 95% CI, 1.13–4.08) and CVD (HR, 1.62; 95% CI, 1.05–2.51). Similar results were obtained when PPG_4–7.9h_ was treated as a dichotomous variable using the top decile as the cutoff (Figure 4). The use of the top decile as the cutoff is based on the estimate from the World Health Organization that 8.5% of adults have diabetes [4]. Subgroup analyses showed that similar results were obtained in those participants without a prior diagnosis of myocardial infarction or stroke (Figure 5).

**Figure 6:**
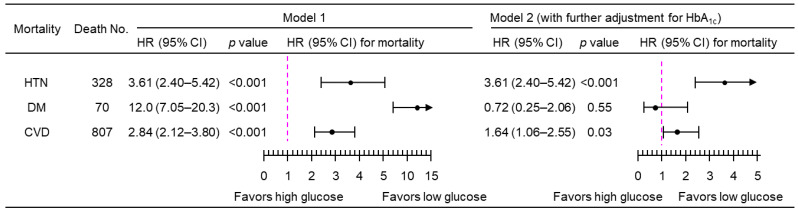
Sensitivity analysis of mortality risk associated with a 1-natural-log-unit increase in PPG_4–7.9h_ in 4781 participants when the imputed data were not used. Model 1: adjusted for age, sex, ethnicity, body mass index, education, poverty income ratio, survey period, physical activity, alcohol consumption, smoking status, systolic blood pressure, total cholesterol, HDL cholesterol, family history of diabetes, and fasting time. Model 2: adjusted for all the factors in Model 1 plus HbA_1c_. CI, confidence interval; CVD, cardiovascular disease; DM, diabetes; HbA_1c_, hemoglobin A_1c_; HDL, high-density lipoprotein; HR, hazard ratio; HTN, hypertension; No., number; PPG_4–7.9h_, postprandial plasma glucose measured from blood taken between 4 and 7.9 h.

**Figure 7:**
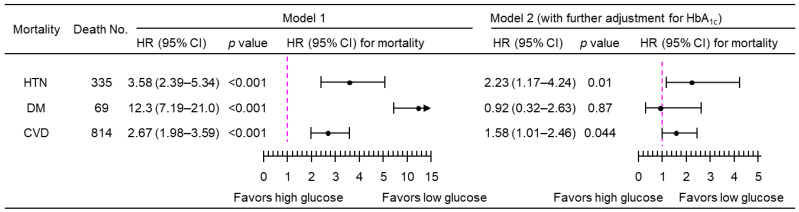
Sensitivity analysis of mortality risk associated with a 1-natural-log-unit increase in PPG_4–7.9h_ in 4851 participants when those with a follow-up time of <1 year were excluded. Model 1: adjusted for age, sex, ethnicity, body mass index, education, poverty income ratio, survey period, physical activity, alcohol consumption, smoking status, systolic blood pressure, total cholesterol, HDL cholesterol, family history of diabetes, and fasting time. Model 2: adjusted for all the factors in Model 1 plus HbA_1c_. CI, confidence interval; CVD, cardiovascular disease; DM, diabetes; HbA_1c_, hemoglobin A_1c_; HDL, high-density lipoprotein; HR, hazard ratio; HTN, hypertension; No., number; PPG_4–7.9h_, postprandial plasma glucose measured from blood taken between 4 and 7.9 h.

**Figure 8:**
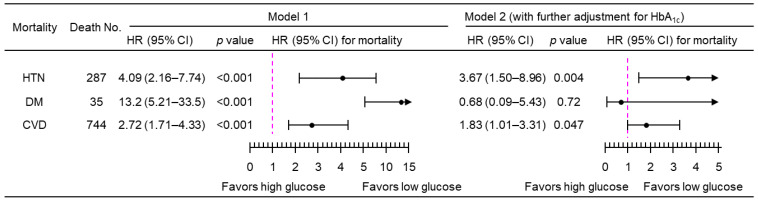
Sensitivity analysis of mortality risk associated with a 1-natural-log-unit increase in PPG_4–7.9h_ in 4646 participants when those who were prescribed with anti-diabetic medications (*n* = 250) were excluded. Model 1: adjusted for age, sex, ethnicity, body mass index, education, poverty income ratio, survey period, physical activity, alcohol consumption, smoking status, systolic blood pressure, total cholesterol, HDL cholesterol, family history of diabetes, and fasting time. Model 2: adjusted for all the factors in Model 1 plus HbA_1c_. CI, confidence interval; CVD, cardiovascular disease; DM, diabetes; HbA_1c_, hemoglobin A_1c_; HDL, high-density lipoprotein; HR, hazard ratio; HTN, hypertension; No., number; PPG_4–7.9h_, postprandial plasma glucose measured from blood taken between 4 and 7.9 h.

Sensitivity analyses showed that PPG_4–7.9h_ remained positively associated with mortality from hypertension and CVD when imputed data were not used, i.e., by excluding those 115 participants with missing data (Figure 6), or when those with a follow-up time of <1 year were excluded (Figure 7), or when those who were prescribed with anti-diabetic medications were excluded (Figure 8).

## 4. Discussion

Using a general cohort of US adults, this study, for the first time, demonstrated that PPG_4–7.9h_ was positively associated with mortality from both hypertension and CVD, independent of HbA_1c_. In addition, these positive associations remained in the sub-cohort of participants who did not have a prior diagnosis of myocardial infarction or stroke.

This study found that PPG_4–7.9h_ was positively associated with hypertension mortality. However, the underlying mechanism is unknown. It is well-known that diabetes and hypertension often co-exist in many individuals [23], and these two conditions share some risk factors such as obesity [24,25] and physical inactivity [26,27]. It has been shown that baseline fasting plasma glucose [28], fasting plasma glucose change trajectory [29], and diabetes [12] are positively associated with risks of hypertension incidence [12], suggesting that high blood glucose may disturb the blood pressure homeostasis. Consistently, the current study showed that PPG_4–7.9h_ was positively associated with hypertension mortality, independent of well-known confounders including body mass index, physical activity, total cholesterol, and HDL cholesterol, supporting a causal role of high plasma glucose in worsening hypertension outcomes. It has been reported that high plasma glucose may lead to oxidative stress and endothelial dysfunction [30,31]. Whether increased oxidative stress and endothelial dysfunction play a role in mediating the positive association between PPG_4–7.9h_ and hypertension mortality needs to the investigated in the future, as does whether lowering PPG_4–7.9h_ is effective in improving blood pressure control and hypertension mortality.

The association of diabetes with CVD incidence and mortality is well documented. Diabetes is an independent risk factor for CVD [32]. In addition, sodium–glucose cotransporter 2 (SGLT2) inhibitors, a class of anti-diabetic medication, decrease CVD events and mortality [33,34,35]. The mechanism underlying the association of diabetes with CVD events and mortality is not well understood.

A few studies have investigated the association of PPG with cardiovascular events. PPG at 1 or 2 h after breakfast [36,37] or 2 h after lunch [38,39] were reported to be positively associated with CVD events. However, those studies did not investigate CVD mortality. In addition, measuring glucose at 1 or 2 h after a meal may not be ideal, as variation in diet could change PPG by more than 20 mg/dL [11], and variation in blood collection time (±0.5 h in practice [40]) could introduce bias as PPG is time sensitive around 1 to 2 h [11]. In contrast, the current study showed that PPG_4–7.9h_ was stable and hourly PPG_4–7.9h_ was comparable. Therefore, PPG_4–7.9h_ may more reliably reflect one’s true ability to control blood glucose after a meal. Whether PPG_4–7.9h_ is superior to PPG at 1 or 2 h after a meal in predicting cardiovascular events needs to be investigated in the future.

Only one study investigated PPG and CVD mortality, which found that PPG measured from blood taken between 3 and 7.9 h was positively associated with CVD mortality [10]. However, the use of PPG measured from blood taken between 3 and 7.9 h is inferior to PPG_4–7.9h_, as PPG measured from blood taken between 3 and 3.9 h did not return to the baseline level and it was higher than PPG_4–7.9h_ [10]. In addition, PPG returned to baseline four hours after a meal regardless of meal type and mealtime [11]. Moreover, the current study confirmed that hourly PPG_4–7.9h_ was similar across the duration from 4 to 7.9 h. Therefore, it is necessary to investigate the association between PPG_4–7.9h_ and CVD mortality.

Some studies have investigated the association between fasting plasma glucose and CVD mortality and the results are inconsistent: some show a positive association [41,42], whereas others show no association [43,44]. The reason for this inconsistency is unknown. This may be due to poor reproducibility of fasting plasma glucose [45]. For instance, only 75% of adults were classified into the same diabetes category (normal, prediabetes, or diabetes) based on two consecutive measures of fasting plasma glucose which were conducted 6 weeks apart [45].

The current study showed that PPG_4–7.9h_ was positively and independently associated with CVD mortality, and such a positive association remained in those without a prior diagnosis of myocardial infarction or stroke. Given its stability and reproducibility_,_ PPG_4–7.9h_ may be a better predictor of CVD mortality than fasting plasma glucose and PPG measured from blood taken between 3 and 7.9 h. Whether lowering PPG_4–7.9h_ is a primary prevention strategy to decrease CVD mortality needs to be investigated in the future.

This study found that PPG_4–7.9h_ was positively associated with diabetes mortality, and such an association disappeared after future adjustment for HbA_1c_, suggesting that HbA_1c_ could explain the association between PPG_4–7.9h_ and diabetes mortality. The underlying mechanism is unknown. HbA_1c_ is a type of hemoglobin that is chemically linked to a sugar and its formation indicates the presence of excessive sugar in the blood. Therefore, HbA_1c_ is an indirect measure of the average blood glucose levels [46] which reflect the blood sugar level over the past 90 days [47]. HbA_1c_ is a good measure of glycemic control [48]. It has been shown that HbA_1c_ is a strong predictor for diabetic ketoacidosis, and adult diabetic patients with an HbA_1c_ of ≥9% have a 12-fold higher incidence of diabetic ketoacidosis than those with an HbA_1c_ of <7% [49]. When diabetes is listed as the leading cause of mortality (i.e., diabetes mortality in the current study), the death more likely results from a glycemic crisis due to diabetic ketoacidosis or a coma. Therefore, HbA_1c_ and PPG_4–7.9h_ may be equally sufficient in differentiating those who have a high risk of fatal glycemic crisis from those with a low risk. Consequently, adjusting HbA_1c_ may diminish the association between PPG_4–7.9h_ and diabetes mortality. This hypothesis needs to be tested in the future.

Some guidelines have started to recommend non-fasting lipids (triglycerides and various forms of cholesterol) as the standard for cardiovascular risk assessment [50,51]. Consistently, the current study suggests that non-fasting plasma glucose (PPG_4–7.9h_) may be used for cardiovascular risk assessment. The non-fasting plasma glucose test is more convenient than a fasting glucose test. Fasting tests are inconvenient, as patients need to present to the laboratory in the morning before eating or drinking, and they likely need to wait a long time while fasting [50]. Patients with diabetes on antidiabetic medications are at risk of developing hypoglycemia when fasting for laboratory testing [52]. In addition, prolonged fasting may be associated with an increased risk of hypoglycemia in those who are frail [50]. In contrast, a non-fasting blood test is more convenient and comfortable for patients and most tests could be performed on the same day of the clinical visit. Therefore, testing non-fasting plasma glucose is more desirable for patients than testing fasting plasma glucose. However, more research is needed to establish whether non-fasting plasma glucose could be eventually used in the clinic for CVD risk assessment. For example, studies replicating the results of the current study using different populations from different countries are needed.

**Strengths and limitations** One strength of this study is its analysis of PPG after meals of free choice in a large representative cohort of US adults. Another strength is its prospective study design with a long follow-up (mean, 21.7 years). A third strength is its adjustment for a large number of confounders. This study also has several limitations. First, mortality outcomes were ascertained by linkage to the National Death Index (NDI) records with a probabilistic match, which could result in misclassification [53]. However, this matching method has been shown to be highly accurate (accuracy, 98.5%) [54]. Second, PPG was only measured at one timepoint for each participant, which may lead to bias. Nevertheless, in epidemiological analysis, this bias tends to result in an underestimate rather than an overestimate of risk due to regression dilution [55]. Therefore, the current study may underestimate the association of PPG_4–7.9h_ with CVD mortality and hypertension mortality. In other words, the association of PPG_4–7.9h_ with CVD mortality and hypertension mortality may be much stronger if repeated PPG_4–7.9h_ measurements were used.

## 5. Conclusions

This study found that PPG_4–7.9h_ is positively associated with mortality from hypertension and CVD, and such positive associations remain in those without a prior diagnosis of myocardial infarction or stroke. Therefore, lowering PPG_4–7.9h_ may be a primary prevention strategy to decrease CVD mortality. PPG_4–7.9h_ may need to be closely monitored in those with an increased CVD risk, in particular in those with hypertension.

## Figures and Tables

**Table 1:** Baseline characteristics of the participants, stratified by the top decile versus the bottom nine deciles of PPG_4–7.9h._

Variables	Bottom 9 Deciles	Top Decile	Overall	*p* Value
Sample size	4408	488	4896	N/A
PPG_4–7.9h_, mg/dL, median (IQR)	91 (86–96)	125 (114–179)	92 (87–99)	<0.001
HbA_1c_, %, median (IQR)	5.3 (5.0–5.6)	6.9 (5.7–8.8)	5.4 (5.0–5.7)	<0.001
BMI, kg/m^2^, median (IQR)	26 (23–30)	28 (25–32)	26 (23–30)	<0.001
SBP, mm Hg, median (IQR)	123 (112–137)	136 (124–152)	124 (113–139)	<0.001
Total cholesterol, mg/dL, median (IQR)	203 (176–234)	218 (191–248)	205 (177–236)	<0.001
HDL cholesterol, mg/dL, median (IQR)	50 (41–60)	46 (38–58)	49 (41–60)	<0.001
Age, y, mean (SD)	48 (18)	61 (18)	49 (19)	<0.001
Fasting time, h, mean (SD)	6.6 (0.8)	6.6 (0.8)	6.6 (0.8)	0.21
Sex (male), *n* (%)	2016 (45.7)	242 (49.6)	2258 (46.1)	0.11
Ethnicity, *n* (%)				<0.001
Non-Hispanic white	2098 (47.6)	200 (41.0)	2298 (46.9)	
Non-Hispanic black	1041 (23.6)	102 (20.9)	1143 (23.3)	
Mexican-American	1099 (24.9)	168 (34.4)	1267 (25.9)	
Other	170 (3.9)	18 (3.7)	188 (3.8)	
Education, *n* (%)				<0.001
<High School	1657 (37.6)	290 (59.4)	1947 (39.8)	
High School	1372 (31.1)	111 (22.7)	1483 (30.3)	
>High School	1349 (30.6)	85 (17.4)	1434 (29.3)	
Unknown	30 (0.7)	2 (0.4)	32 (0.7)	
Poverty income ratio, *n* (%)				<0.001
<130%	1167 (26.5)	178 (36.5)	1345 (27.5)	
130–349%	1834 (41.6)	179 (36.7)	2013 (41.1)	
≥350%	1077 (24.4)	74 (15.2)	1151 (23.5)	
Unknown	330 (7.5)	57 (11.7)	387 (7.9)	
Physical activity, *n* (%)				<0.001
Active	1634 (37.1)	136 (27.9)	1770 (36.2)	
Insufficiently active	1863 (42.3)	213 (43.6)	2076 (42.4)	
Inactive	911 (20.7)	139 (28.5)	1050 (21.4)	
Alcohol consumption, *n* (%)				<0.001
0 drink/week	755 (17.1)	126 (25.8)	881 (18.0)	
<1 drink/week	503 (11.4)	37 (7.6)	540 (11.0)	
1–6 drinks/week	857 (19.4)	55 (11.3)	912 (18.6)	
≥7 drinks/week	555 (12.6)	50 (10.2)	605 (12.4)	
Unknown	1738 (39.4)	220 (45.1)	1958 (40.0)	
Smoking status, *n* (%)				<0.001
Past smoker	1086 (24.6)	87 (17.8)	1173 (24.0)	
Current smoker	1109 (25.2)	182 (37.3)	1291 (26.4)	
Other	2213 (50.2)	219 (44.9)	2432 (49.7)	
Survey period, *n* (%)				0.57
1988–1991	2168 (49.2)	247 (50.6)	2415 (49.3)	
1991–1994	2240 (50.8)	241 (49.4)	2481 (50.7)	
Family history of diabetes, *n* (%)				<0.001
Yes	1911 (43.4)	261 (53.5)	2172 (44.4)	
No	2420 (54.9)	217 (44.5)	2637 (53.9)	
Unknown	77 (1.7)	10 (2.0)	87 (1.8)	

Abbreviations: BMI, body mass index; HbA_1c_, hemoglobin A_1c_; HDL, high-density lipoprotein; IQR, interquartile range; *n*, number; N/A, not applicable; PPG_4–7.9h_, postprandial plasma glucose measured from blood taken between 4 and 7.9 h; SBP, systolic blood pressure; SD, standard deviation; y, year.

## Data Availability

All data in the current analysis are publicly available on the NHANES website (https://www.cdc.gov/nchs/nhanes/index.htm), accessed on 10 February 2022.
